# Immunoglobulin G4-related autoimmune hepatitis with overlapping multiple autoimmune diseases: A case report

**DOI:** 10.1097/MD.0000000000043630

**Published:** 2025-07-25

**Authors:** Jun Long, Yuchen Shi, Qiuyan Yao, Xiaona Zhou, Meihua Luo, Rongjie Shi

**Affiliations:** a The Clinical Medical College of Dali University, Dali, Yunnan Province, China; b First Affiliated Hospital of Kunming Medical University, Kunming, Yunnan Province, China; c Department of Gastroenterology, the First Affiliated Hospital of Dali University, Dali, Yunnan Province, China.

**Keywords:** case report, IgG4-related autoimmune hepatitis, monoclonal gammopathy of undetermined significance, primary biliary cholangitis, Sjogren syndrome

## Abstract

**Rationale:**

The prevalence of autoimmune liver disease in Sjogren syndrome (SS) ranges from 1.7% to 47%. Hematologic involvement is commonly observed in SS patients, with 1.5% of patients presenting with monoclonal gammopathy of undetermined significance (MGUS). Chronic antigenic stimulation of B lymphocytes in autoimmune diseases may eventually lead to clonal proliferation or MGUS. Nevertheless, studies reporting the concurrent manifestation of IgG4-related autoimmune hepatitis (IgG4-AIH), Primary biliary cholangitis (PBC), SS, and MGUS are scarce.

**Patient concerns:**

This article reports a rare case involving the overlap of multiple autoimmune diseases. A middle-aged female patient experienced fatigue, anorexia, xerostomia, jaundice, and pruritus, as well as elevated levels of liver enzymes, globulin, and monoclonal immunoglobulin (M protein).

**Diagnoses:**

Based on the findings of serum IgG4 levels, autoimmune serology, liver and labial gland biopsy, and bone marrow puncture, a diagnosis of IgG4-AIH, PBC, SS, and MGUS was made.

**Interventions:**

The patient was initiated on oral prednisone 40 mg once daily and ursodeoxycholic acid capsules 250 mg 3 times daily. Upon stabilization of the condition, prednisone was progressively tapered to a maintenance dose of 5 mg daily.

**Outcomes:**

Over the 6-month follow-up period, favorable improvements were noted in the patient’s symptoms.

**Lessons:**

The purpose of this report’s research is to thoroughly describe the clinical manifestations, diagnostic challenges, and treatment difficulties associated with the rare co-existence of multiple autoimmune diseases and hematological diseases, and to summarize the management methods. The significance of this study lies in emphasizing the importance of screening for autoimmune diseases and hematological diseases in patients presenting similar symptoms in clinical practice, aiming to provide a reference for clinicians in identifying and managing such complex cases, thereby enhancing the understanding and treatment level of these diseases.

## 1. Introduction

Autoimmune hepatitis (AIH) and primary biliary cholangitis (PBC) are autoimmune liver diseases characterized by the presence of specific autoantibodies and histopathological features. Both conditions may co-exist simultaneously or successively, a phenomenon referred to as overlap syndrome (OS). According to a previous study, the prevalence of AIH-PBC overlap among patients with autoimmune liver disease is approximately 12 %.^[1]^ IgG4-related disease (IgG4-RD) is a chronic fibroinflammatory autoimmune disease involving multiple systems and organs. It is hallmarked by elevated serum IgG4 levels and the enlargement of affected tissues and organs. Histopathologically, IgG4-RD is characterized by the infiltration of IgG4-positive plasma cells into tissues and organs, storiform fibrosis, occlusive phlebitis, and sensitivity to steroid therapy.^[2]^ Hepatic infiltration by IgG4-positive plasma cells can lead to IgG4-related liver disease and IgG4-related autoimmune hepatitis. The latter is a rare disease that requires meeting the diagnostic criteria for both IgG4-RD and AIH, accompanied by the infiltration of IgG4-positive plasma cells into hepatic tissues. Notably, the involvement of salivary glands can lead to IgG4-related sialadenitis (IgG4-RS), which shares clinical features similar to Sjogren syndrome, encompassing glandular enlargement, xerostomia, and arthralgia. However, the prevalence of autoimmune antibodies is lower in patients with IgG4-RS compared to those with SS.^[3]^ As is well documented, the prevalence of autoimmune liver disease in SS patients ranges from 1.7 % to 47 %.^[4–6]^ Moreover, hematologic involvement is generally noted in SS patients, with 1.5 % of SS patients presenting with monoclonal gammopathy of undetermined significance (MGUS). Chronic antigenic stimulation of B lymphocytes in autoimmune diseases may eventually culminate in clonal proliferation or MGUS.^[7]^ Nevertheless, studies reporting the co-existence of IgG4-AIH, PBC, SS, and MGUS are limited. This report outlines the case of a 58-year-old Chinese woman diagnosed with 4 conditions, namely IgG4-AIH, PBC, SS, and MGUS. The patient was admitted due to unexplained liver function damage. Treatment with ursodeoxycholic acid and prednisone improved liver function, following which the patient was discharged.

## 2. Patient concerns

A 58-year-old Chinese woman presented with a 1-year history of unexplained fatigue, anorexia, and xerostomia, accompanied by generalized jaundice and pruritus. She had been treated at the outpatient clinic of the hospital with ursodeoxycholic acid and polyene phosphatidylcholine capsules. Although jaundice subsided, liver function indices remained elevated. She was re-admitted due to liver function damage. The patient had no history of chronic liver disease, alcohol use, blood transfusion, or viral hepatitis. In addition, she did not report the use of hepatotoxic drugs or herbal products. During the physical examination, she was alert and oriented. There was no evidence of conjunctival pallor or scleral and cutaneous icterus. Likewise, respiratory and cardiovascular examinations were unremarkable. The abdomen was soft and non-tender. Murphy sign was negative. No neurological impairment or asterixis was noted.

### 2.1. Admission laboratory examination

Laboratory investigations revealed a white blood cell count of 3.43 * 10^9^/L, a red blood cell count of 3.48 * 10^12^/L, and hemoglobin and platelet counts were within the normal range. At the same time, serum biochemistry showed a total bilirubin level of 20.6 µmol/L, direct bilirubin of 11 µmol/L, alanine aminotransferase of 66 U/L, aspartate aminotransferase of 108 U/L, alkaline phosphatase of 231 U/L, gamma-glutamyl transpeptidase of 241 U/L, total protein of 107.9 g/L, globulin of 83.5 g/L, total bile acid of 17.7 µmol/L, and serum calcium of 2.21 mmol/L, as listed in Table 1. Immunoglobulin profiling demonstrated an immunoglobulin G of 65.4 g/L, an immunoglobulin A level of 4.24 g/L, and an immunoglobulin M level of 4.14 g/L. Antinuclear antibody screening revealed antinuclear antibody > 400.00 RU/mL, anti-SSA antibody > 400.00 RU/mL, anti-Ro-52 > 400.00 RU/mL, anti-SSB antibody > 400.00 RU/mL, and anti-mitochondrial-M2 antibody > 400.00 RU/mL. Furthermore, immunoglobulin G4 > 14 g/L. Testing for Epstein–Barr virus and viral hepatitis yielded negative results. Finally, routine analyses of urine, stool, fecal occult blood, tumor markers, thyroid function, coagulation function, anticardiolipin antibodies, vascular endothelial growth factor, and electromyography were unremarkable.

**Table 1:** Laboratory results before and after admission.

Hepatic function parameter	Hospitalization 1 yr ago	Admission	1 mo after discharge	2 mo after discharge	6 mo after discharge	2 yr after discharge	Reference range
ALT	135	66	68	87	25	29	0–50 U/L
AST	222	108	118	104	40	65	0–50 U/L
ALP	323	231	230	162	126	172	40–150 U/L
GGT	399	241	149	117	85	68	11–50 U/L
ALB	26.4	24.4	19	29.6	32.4	23.1	35–55 g/L
GLB	84.9	83.5	64.6	57.6	52.5	46.4	20–40 g/L
TBI	17.7	20.6	21.7	23.1	22.5	17.2	5.1–19 µmmol/L
DBI	9.7	11.0	15.3	13.6	8.6	8.7	0–5.1 µmmol/L

ALB = albumin, ALP = alkaline phosphatase, ALT = alanine aminotransferase, AST = aspartate aminotransferase, DBI = direct bilirubin, GLB = globulin, TBI = total bilirubin.

### 2.2. Imaging examination

**Figure 1.**
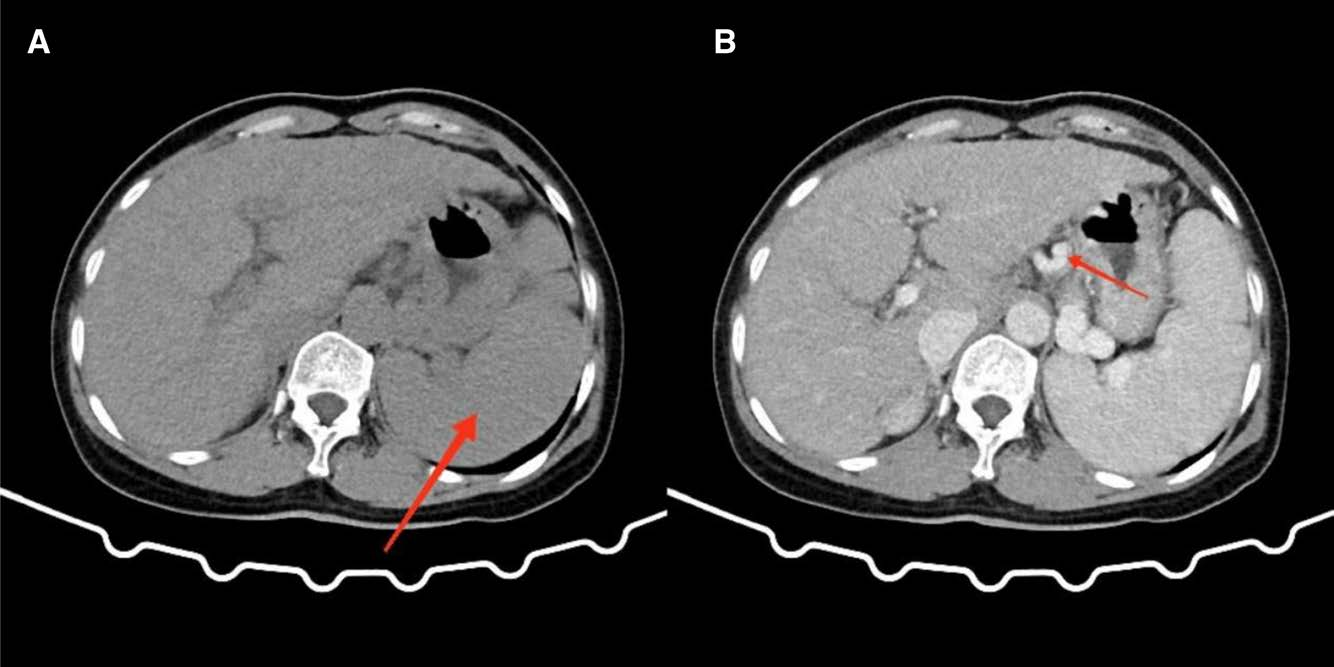
Upper plain and contrast-enhanced abdominal CT scans. (A) Red arrow indicates splenomegaly. (B) The portal venous phase CT, with the red arrow pointing towards gastric varices.

Abdominal CT (both plain and contrast-enhanced scans) displayed cirrhosis, splenomegaly, severe portal hypertension with esophageal-gastric fundus varices, as well as additional varicose changes (see Fig. 1). Chest CT illustrated mild chronic inflammation in the lower lobe of both lungs. Thoracolumbar spine X-rays in anteroposterior and lateral views suggested degenerative osteoarthropathy. No major abnormalities were identified on color Doppler ultrasound of superficial lymph nodes throughout the body and the X-rays of the skull.

### 2.3. Pathological findings

**Figure 2.**
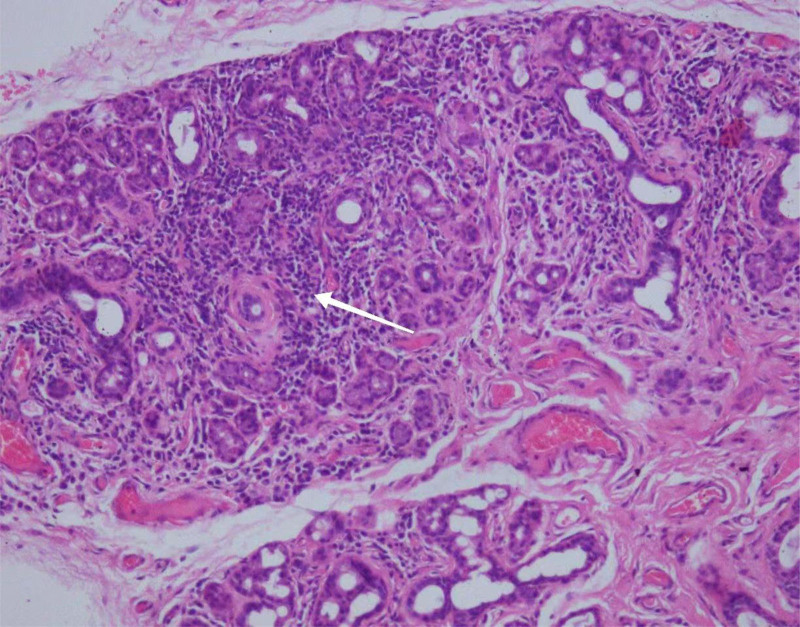
Labial gland biopsy: arrow indicates focal lymphocytes, HE staining (×200).

**Figure 3.**
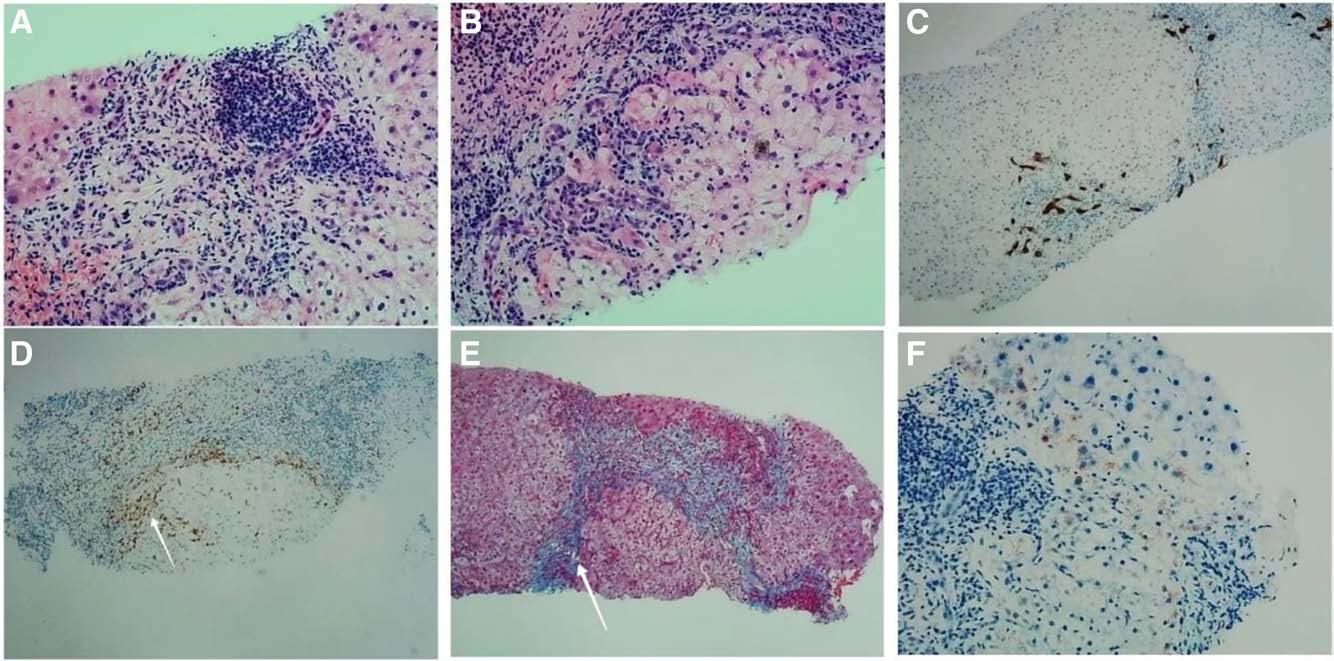
Pathological examination of liver tissue. (A) moderate interface hepatitis, HE staining (x200). (B) Bridging fibrosis, accompanied by local pseudolobule formation, HE staining (×200). (C) Positive CK19 staining in bile duct epithelial cells with mild bile duct hyperplasia, immunohistochemical staining (×100). (D) An increased proportion of plasma cells, IgG (+, >100/HPF), IgG4 (+, 11/HPF), arrows indicate IgG-positive cells, immunohistochemical staining (×100). (E) arrows represent fibrosis, Masson staining (×100), (F) copper staining (×100).

Labial gland biopsy showed multifocal lymphocytic infiltration between acinar cells, consistent with Sjogren syndrome (see Fig. 2). Liver biopsy showed features of autoimmune hepatitis and PBC (AIH + PBC), IgG4-related hepatitis, chronic active hepatitis, moderate inflammation, and moderate to severe fibrosis. The modified score Scheuer score was G3S3–4 (see Fig. 3). As delineated in Figure. A, moderate interface hepatitis with lymphocyte and plasma cell infiltration, accompanied by occasional neutrophils and eosinophils, was observed in the portal area. Figure. B indicates that the structure of hepatic lobules is disordered. As anticipated, the portal area was enlarged to varying degrees, accompanied by fibrous tissue proliferation, bridging fibrosis, chronic inflammatory cell infiltration, and the formation of localized pseudolobules. Figure. C depicts positive CK19 staining in the bile duct epithelium and mild bile duct hyperplasia. Figure D shows an increased proportion of MUM1-positive plasma cells, IgG (+, > 100/ HPF), and IgG4 (+, 11/ HPF). Figure E shows the presence of bridging fibrosis and local pseudolobule formation. Lastly, Figure. F shows the deposition of copper particles in approximately 5% of hepatocytes around the portal area.

### 2.4. Further examination

Serum IgG levels were significantly elevated (>4 times ULN), prompting further auxiliary examination to investigate the possibility of an underlying hematological disease. Serum immunofixation electrophoresis revealed an M protein concentration of 19.90 g/L, with the M type identified as IgG-K. Assessment of serum-free light chains demonstrated a serum-free Kappa light chain level of 389.31 mg/L, a serum-free lambda light chain of 88.61 mg/L, and a serum-free Kappa/free lambda ratio of 4.3935. Furthermore, serum protein electrophoresis revealed a distribution of albumin at 27.2%, α1-globulin at 2.0%, α2-globulin at 5.4%, β1-globulin at 3.4%, β2-globulin at 4.1%, γ-globulin at 57.9 %, an M-protein proportion of 21.4 %, and the presence of M protein bands. Urine Bence-Jones protein testing yielded negative results. Besides, bone marrow aspiration of the left posterior superior iliac spine uncovered that plasma cells accounted for 8.5% of cells, largely comprising immature and mature plasma cells, with red blood cells evenly arranged. Given the high proportion of plasma cells, a repeat aspiration was performed from the right posterior superior iliac spine. The smear revealed active bone marrow hyperplasia, with a granulocyte/erythroid ratio of 2.68:1. Interestingly, the 3 hematopoietic lineages were active, accompanied by scattered platelets. Plasma cells accounted for 5.5% of all cells, and occasional binucleated plasma cells were observed. Flow cytometry of the bone marrow showed the presence of roughly 1.21% monoclonal plasma cells. Consequently, the possibility of plasma cell disease was considered.

## 3. Diagnosis

Finally, a diagnosis of IgG4-AIH, PBC, SS, and MGUS was established.

## 4. Interventions

The patient, weighing 52 kg, was maintained on oral ursodeoxycholic acid at a dose of 250 mg 3 times daily, owing to the diagnosis of IgG4-AIH overlapping with PBC and SS. Given that the results of abdominal CT and liver biopsy suggested moderate to severe liver fibrosis and cirrhosis, the patient was initiated on oral prednisone at a daily dose of 40 mg. Importantly, the clinical symptoms of the patients were alleviated following treatment. Concomitantly, liver function parameters improved, and thus the patient was discharged. Upon achieving clinical stability, the patient was instructed to gradually taper prednisone to 5 mg daily.

## 5. Outcomes

After the patient took ursodeoxycholic acid capsules and prednisone tablets for 1 month, symptoms such as fatigue, skin itching, and dry mouth gradually improved. However, liver enzymes remained high. Therefore, we did not adjust the dose of prednisone temporarily. We instructed the patient to continue oral administration and schedule a follow-up appointment for 1 month. After taking the medication for 2 months, the patient’s symptoms significantly improved, and liver enzymes also decreased. We informed the patient to start reducing the prednisone dose by 5 mg every 2 weeks, gradually reducing it to a long-term maintenance dose of 5 mg. However, during the reduction period, it is essential to check liver function regularly. After 6 months of outpatient visits and telephone follow-up, symptoms such as fatigue, anorexia, pruritus, and xerostomia had resolved, while the levels of transaminases, cholestatic enzymes, and globulin significantly decreased (see Table 1). The patient remains under active follow-up. Before the release of anonymized data, written informed consent was obtained from the patient.

## 6. Discussion

IgG4-related disease is a chronic, fibroinflammatory autoimmune disease hypothesized to be related to the activation of numerous types of immune-mediated lymphocytes. It is hallmarked by an elevated proportion of IgG4-positive plasma cells in serum and swelling of affected tissues and organs. Histopathological features encompass the infiltration of IgG4-positive plasma cells into tissues and organs, storiform fibrosis, and occlusive phlebitis. Of note, it is sensitive to steroid therapy and can target multiple tissues and organs, such as the lacrimal gland, parotid gland, liver, pancreas, and biliary tract.^[2]^ IgG4-associated autoimmune hepatitis can be caused by IgG4-positive plasma cells infiltrating the liver. In 2007, Umemura et al^[8]^ first reported a middle-aged female patient with IgG4-AIH, leading to the proposition of IgG4-AIH as a novel disease subtype. In 2010, the research team reported another case of IgG4-AIH,^[9]^ which has since garnered extensive attention. IgG4-AIH is a rare disease that must concurrently meet the diagnostic criteria of IgG4-RD and autoimmune hepatitis. The diagnosis is typically established by the presence of IgG4-positive plasma cell infiltration into the liver. At present, there is no universally established diagnostic criteria for IgG4-AIH. Umemura et al proposed the following diagnostic criteria^[10]^: presence of AIH; serum IgG4 level ≥ 135 mg/dL; IgG4-positive plasma cells infiltration into liver tissue (≥10/HPF).

While autoimmune hepatitis and PBC are both autoimmune liver diseases, their clinical manifestations and pathophysiological features differ. In rare cases, 2 or more diseases may co-exist either simultaneously or successively, a condition termed overlap syndrome. Noteworthily, AIH-PBC OS is the more common form of OS among autoimmune liver diseases. The diagnostic criteria currently adopted for the diagnosis of AIH-PBC OS are based on the Paris criteria, with diagnostic components for AIH including: (1) ALT levels > 5 times ULN, (2) IgG levels > 2 times ULN or ASMA-positivity, (3) evidence of lymphocytic infiltration in the portal and periportal regions, accompanied by moderate or severe interface hepatitis. The criteria for PBC components include (1) ALP level > 2 times ULN or γ-GGT level > 5 times ULN, (2) AMA-positivity, and (3) liver biopsy suggestive of nonsuppurative cholangitis and intrahepatic bile duct destruction. The diagnosis of AIH-PBC OS must meet at least 2 criteria for each component.^[11]^ Recent studies have documented that the incidence of AIH-PBC OS in patients with autoimmune liver disease ranges between 5.7% and 12%.^[1]^ The long-term combination of ursodeoxycholic acid and glucocorticoids is recommended for the management of AIH-PBC OS.^[12]^

The patient described in this report fulfilled the diagnostic criteria for AIH, and serum IgG4 levels were significantly increased. Liver biopsy suggested the infiltration of IgG4-positive plasma cells, further supporting the diagnosis of IgG4-AIH. In addition, the levels of cholestatic liver enzymes, namely ALP and γ-GGT, were increased, and the patient tested positive for AMA-M2 antibodies. Furthermore, liver biopsy revealed mild bile duct injury. The patient had a prior history of jaundice, which resolved following treatment with ursodeoxycholic acid. However, imaging did not display evidence of IgG4-related sclerosing cholangitis (IgG4-SC), such as bile duct stenosis and deformation. Therefore, the possibility of IgG4-SC was excluded, and a diagnosis of PBC was considered. It is worthwhile emphasizing that the patient met the diagnostic criteria for both IgG4-AIH and PBC. To diagnose overlap syndrome more accurately, the Paris criteria were applied, where the diagnostic criteria for AIH-PBC OS were also met. Therefore, a diagnosis of IgG4-AIH with comorbid PBC was established. The prevalence of AIH-PBC OS is relatively low, and this case represents a rare instance of IgG4-AIH and PBC overlap. To date, no studies have reported the prevalence of such an overlap. Considering that the first reported case of IgG4-AIH progressed to IgG4-SC during the follow-up period despite standardized diagnosis and treatment, ongoing monitoring and follow-up will be performed to determine whether the present patient with PBC will progress to IgG4-SC in the future.

Given that autoimmune liver disease is triggered by autoimmune dysfunction and liver damage, their co-existence with other autoimmune diseases, such as rheumatoid arthritis and Sjogren syndrome, is typically accompanied by extrahepatic manifestations, such as joint pain, xerophthalmia, xerostomia, and pruritus. Moreover, a recent Mendelian randomization study concluded that autoimmune liver disease is associated with an increased risk of Sjogren syndrome.^[13]^ Therefore, given the presence of xerostomia and positive autoimmune antibodies, we screened for Sjogren syndrome. However, IgG4-RD generally involves the salivary glands, which can lead to IgG4-related sialadenitis. IgG4-RS shares clinical symptoms similar to Sjogren syndrome, such as glandular enlargement, sicca symptoms, arthralgia, etc. However, autoimmune antibodies are less commonly detected in patients with IgG4-RS compared to those with SS. Herein, histopathological examination of labial gland tissue revealed multifocal lymphocytic infiltration and no evidence of salivary gland enlargement, which was consistent with Sjogren syndrome. Thus, a diagnosis of SS was made.

Earlier studies have evinced that autoimmune diseases are associated with an increased risk of malignancies,^[14–17]^ which may be ascribed to chronic activation of the immune system. However, the underlying pathological mechanism remains elusive. Prior investigations have outlined that autoimmune diseases are associated with a significantly higher mortality risk by 1.4-fold in MGUS patients and with an increased risk of multiple myeloma, suggesting a shared genetic susceptibility between autoimmune diseases and plasma cell diseases.^[18]^ The pathogenesis of autoimmune diseases and MGUS is complex. Earlier studies have suggested that chronic inflammation associated with autoimmune diseases may contribute to the development of MGUS, whilst chronic antigenic stimulation of B lymphocytes may eventually lead to clonal proliferation, which in turn leads to MGUS or Multiple myeloma. Herein, the patient’s IgG level was significantly increased. To exclude the possibility of hematological disease, further investigations were conducted, revealing the presence of monoclonal immunoglobulin in the patient’s serum. Therefore, bone marrow puncture and imaging examination were carried out, and the results indicated the absence of terminal organ damage. Finally, a diagnosis of MGUS was made.

Currently, the recommended treatment for AIH-PBC OS patients consists of long-term or lifelong administration of ursodeoxycholic acid combined with glucocorticoids.^[12]^ The first-line treatment for IgG4-AIH is corticosteroids,^[19]^ with the initial dose ranging from 0.6 to 0.8 mg/(kg·d). Upon achieving disease control, the dosage can be gradually tapered by 5 mg every 1 to 2 weeks until a maintenance dose is reached. Although corticosteroids provide a high rate of initial remission after short-term immunosuppressive therapy, relapses and flare-ups remain common. Previous studies have reported that the first case of IgG4-AIH achieved normalization of liver enzyme levels after systematic and standardized treatment. Nevertheless, disease progression to IgG4-SC occurred after 5 years.^[20]^ Therefore, despite the risks of osteoporosis, diabetes, and infections associated with long-term use of corticosteroids, it is recommended to maintain low-dose corticosteroids (5 mg) for 3 years or longer to mitigate the risk of recurrence.^[21]^ Therefore, this patient continued to receive ursodeoxycholic acid combined with glucocorticoids. Moreover, she was instructed to ensure rest, avoid fatigue, follow an easily digestible diet, optimize nutritional intake, and strictly avoid hepatotoxic drugs. Following discharge, strict compliance with medical guidance and regular follow-up visits every 3 months at the liver disease clinic plays are critical in monitoring the clinical status of the patient. At the time of writing, the patient remains under follow-up and exhibits a favorable therapeutic response.

Our research has certain limitations. Although patients showed significant improvement in symptoms after treatment, the long-term efficacy and prognosis remain unclear. Whether IgG4-AIH and MGUS continue to progress requires further long-term follow-up to evaluate the effectiveness and safety of the treatment methods.

## 7. Conclusions

This case highlights the multi-systemic nature of autoimmune diseases and the potential for their co-existence with malignancies or hematological diseases. The patient was eventually diagnosed with IgG4-AIH, PBC, SS, and MGUS, which is a rare overlap of 4 conditions. The clinical manifestations spanned multiple specialties. Treatment with ursodeoxycholic acid alone yielded limited improvements in liver function indicators. On the other hand, the addition of steroids rapidly normalized liver damage indicators. This rare presentation offers valuable insights into the overlapping manifestations of multiple autoimmune diseases and the selection of pharmacological agents. Finally, it emphasizes the importance of screening for autoimmune and hematological diseases in patients manifesting multisystem involvement.

## Author contributions

**Conceptualization:** Rongjie Shi.

**Data curation:** Qiuyan Yao, Xiaona Zhou.

**Formal analysis:** Jun Long, Yuchen Shi.

**Investigation:** Jun Long, Yuchen Shi.

**Resources:** Jun Long, Yuchen Shi, Qiuyan Yao, Xiaona Zhou, Meihua Luo.

**Writing – original draft:** Jun Long, Yuchen Shi.

**Writing – review & editing:** Jun Long, Rongjie Shi.
